# Understanding the evaluation of mHealth app features based on a cross-country Kano analysis

**DOI:** 10.1007/s12525-020-00455-y

**Published:** 2021-03-24

**Authors:** Henner Gimpel, Tobias Manner-Romberg, Fabian Schmied, Till J. Winkler

**Affiliations:** 1 University of Hohenheim, Schloss Hohenheim 1, 70599 Stuttgart, Germany; 2 FIM Research Center, University of Augsburg, Universitaetsstr. 12, 86159 Augsburg, Germany; 3 Project Group Business & Information Systems Engineering, Fraunhofer FIT, Universitaetsstr. 12, 86159 Augsburg, Germany; 4 University of Hagen, Universitaetsstr. 47, 58097 Hagen, Germany; 5 Copenhagen Business School, Howitzvej 60, 2000 Frederiksberg, Denmark

**Keywords:** Personal health record, Kano model, Privacy concerns, mHealth literacy, mHealth self-efficacy, Adult playfulness, I10, I12, I18, M3, O3

## Abstract

While mobile health (mHealth) apps play an increasingly important role in digitalized health care, little is known regarding the effects of specific mHealth app features on user satisfaction across different healthcare system contexts. Using personal health record (PHR) apps as an example, this study identifies how potential users in Germany and Denmark evaluate a set of 26 app features, and whether evaluation differences can be explained by the differences in four pertinent user characteristics, namely privacy concerns, mHealth literacy, mHealth self-efficacy, and adult playfulness. Based on survey data from both countries, we employed the Kano method to evaluate PHR features and applied a quartile-based sample-split approach to understand the underlying relationships between user characteristics and their perceptions of features. Our results not only reveal significant differences in 14 of the features between Germans and Danes, they also demonstrate which of the user characteristics best explain each of these differences. Our two key contributions are, first, to explain the evaluation of specific PHR app features on user satisfaction in two different healthcare contexts and, second, to demonstrate how to extend the Kano method in terms of explaining subgroup differences through user characteristic antecedents. The implications for app providers and policymakers are discussed.

## Introduction

Mobile health applications (mHealth apps) play an increasingly important role in the digitalization of nationwide healthcare services for better health outcomes due to the ubiquity of smartphones in society (Ali et al., 2016; Bhavnani et al., 2016; Birkhoff & Moriarty, 2020; Messner et al., 2019; Stoyanov et al., 2015; Xu & Liu, 2015). In 2017, the number of available mHealth apps was estimated at approximately 300,000 and will grow by about 25% every year (Benjumea et al., 2020; Larson, 2018). Frequent examples of mHealth apps are disease-specific apps (e.g., for diabetes), apps for strengthening health competence or adherence (e.g., medication reminders and diet and nutrition tracking), and apps for the storage and exchange of health-related data (e.g., personal health records (PHRs)) (Aitken et al., 2017; Jimenez et al., 2019; Knöppler et al., 2016). The usage of mHealth apps promises excellent opportunities, including improvement in user self-management and user empowerment (Wickramasinghe et al., 2012; Zapata et al., 2015). For example, throughout the COVID-19 pandemic, tracking apps have been used for contact tracing and monitoring infected individuals (Salathé et al., 2020). Moreover, PHR apps are promoted as a digital solution toward greater patient empowerment by integrating health data in one spot (Helmer et al., 2011; Sachverständigenrat Gesundheitswesen, 2020; Schneider et al., 2016). Although literature agrees on the considerable potential of mHealth apps, the current adoption of mHealth apps is still low (de Lusignan et al., 2013; Ozok et al., 2017; Thies et al., 2017). Furthermore, the retention rate of actual mHealth app users is comparatively low (Vaghefi & Tulu, 2019; Zhou et al., 2019b). Due to the plethora of available mHealth apps (Benjumea et al., 2020; Larson, 2018), there is a wide variability in quality and key features of the apps (Jimenez et al., 2019). Because of this abundance, users struggle to identify appropriate, secure, and trustworthy mHealth apps that fulfill their specific needs (Jimenez et al., 2019; van Haasteren et al., 2020). To overcome this challenge, several authors suggest to better involve relevant stakeholders to the app development process (Jimenez et al., 2019; Marent et al., 2018). Within our paper, we focus on mHealth app users as a relevant stakeholder group to better understand their needs and preferences and to contribute to the development of more appropriate apps.

Specific mHealth app features’ relative attractiveness to user groups in different countries is not yet well understood. Despite country-dependent conditions, such as the technological infrastructure and cultural attitudes (Wickramasinghe & Schaffer, 2010), the preponderance of mHealth research has addressed user acceptance of mHealth only on the app level (e.g., Abd-Alrazaq et al., 2019; Bin Azhar & Dhillon, 2016; Dehzad et al., 2014; Stoyanov et al., 2015; Vaghefi & Tulu, 2019). While providing important insight into the factors influencing the general attractiveness of mHealth apps, the app-level approach obscures differences in the feature evaluation of the specific mHealth app, which typically consists of a bundle of privacy-related (Kharrazi et al., 2012), data-related (Maloney & Wright, 2010), functionality-related (e.g., Cabitza et al., 2015), and other possible features, such as gamification (e.g., Mendiola et al., 2015). Furthermore, most prior mHealth research has evaluated mHealth apps in a single geography (e.g., La Torre Díez et al., 2017; Lee & Jung, 2018) and thus has implicitly ignored the potential influences of technological, legal, and cultural variations across countries on the attitudes of the user groups. Feature-specific knowledge about mHealth apps that is sensitive to the potential influence of the country context is valuable to mHealth app providers (e.g., governmental agencies, health insurances, and startups) to provide apps that satisfy the specific user needs and thus to enhance the so-far underwhelming adoption rates of most mHealth apps.

To address the gap in our knowledge on the feature-specific and context-sensitive evaluation of mHealth apps, we focus on the case of the PHR app and the potential users in two countries representing distinct healthcare system contexts in Europe: Germany and Denmark. The PHR apps are a suitable representative of mHealth apps because they cover various features relevant to a broad segment of society (Roehrs et al., 2017). Our focus on German and Danish^1^ users provides an adequate basis for comparative analysis within the European Union. Both countries have a joint background in European regulation and similar Western values, whereas they differ concerning critical aspects of digital health care. While the Danish *Beveridge* health system is often thought of as a digital leader, Germany’s *Bismarck* health system is frequently considered to be at the slower end of the innovation curve (Bertelsmann Stiftung, 2018; Kierkegaard, 2013; Nohl-Deryk et al., 2018; Stroetmann et al., 2011). For example, Denmark launched a nationwide PHR (sundhed.dk) in 2003 (Gherardi et al., 2014), whereas PHR solutions in Germany are still fragmented and not widely adopted (Fitte et al., 2019). Consequently, the two countries represent two different predominant healthcare system types in Europe with different innovation positions. To understand potential differences in the evaluation of PHR features across the two countries, we focus on four pertinent user characteristics that have either been discussed in prior literature as factors influencing mHealth app adoption (privacy concerns, mHealth literacy, and mHealth-self-efficacy) or have been proposed to influence user satisfaction with mHealth apps more generally (adult playfulness).

Thus, we raise the following two research questions:

**RQ1:** How do potential users in Germany and Denmark evaluate a broad set of specific PHR features?

**RQ2:** Do user characteristics (specifically privacy concerns, mHealth literacy, mHealth self-efficacy, and adult playfulness) explain the differences in the evaluation of PHR features by potential users in Germany and Denmark?

To answer the research questions, we identified 26 potential PHR app features from the prior literature. We designed a cross-national survey using the Kano method (for evaluating these features) and assessing user characteristics. The Kano method (Kano et al., 1984) is widely applied in information systems as a suitable method to understand user preferences regarding the specific attributes of a product or service (i.e., the features) as one out of four main categories (attractive, one-dimensional, must-be, or indifferent quality) (Gimpel et al., 2018; Hejaili et al., 2009). To identify possible explanations for evaluation differences between Germans and Danes, we apply a quartile-based sample split on each of the user characteristics and compare the resulting categorizations in the upper and lower quartiles with the categorization differences between Germans and Danes.

Our results from a survey of 274 participants (215 Germans and 59 Danes) demonstrate significant and meaningful differences in the evaluation of features and the evaluation between Germans and Danes. Moreover, given the empirical results that demonstrate significant group differences between Germans and Danes on the four user characteristics, we demonstrate that user characteristics help explain the evaluation differences for 14 of the 26 features. Generally, the findings indicate that users with lower privacy concerns, higher mHealth literacy, higher mHealth self-efficacy, and higher playfulness (such as Danish users) tend to evaluate more PHR features as attractive. In contrast, users with higher privacy concerns, lower mHealth literacy, lower mHealth self-efficacy, and lower playfulness (such as German users) tend to evaluate more PHR features as indifferent. We argue that our study not only explains the evaluation of a broad range of PHR app features across two representative countries but also demonstrates how to methodologically augment the Kano model with an analytical method for explaining emerging subgroup differences using antecedent user characteristics.

In the following sections, we set the theoretical foundations and develop the research hypotheses (Section 2). We also explain the research method (Section 3) and provide empirical results (Section 4). Moreover, we discuss the implications, limitations, and future research (Section 5) and conclude the work (Section 6).

## Theoretical foundations and hypothesis development

This section reviews the theory behind the Kano model. This section also introduces PHRs and their features and develops the research hypotheses regarding the influence of the four user characteristics.

### Kano theory of user satisfaction

The *user satisfaction*^2^ construct is of high relevance in both research and practice due to its influence on consumer behavior (Oliver, 2014). For instance, user satisfaction has a positive impact on user loyalty (Gronholdt et al., 2000) and the overall company value (Stahl et al., 2000). Initially, user satisfaction has often been considered a one-dimensional construct: the higher the perceived product or service quality, the higher the user satisfaction, and vice versa (Yi, 1990). However, solely fulfilling user expectations to a great extent does not necessarily imply a high level of user satisfaction; it is also the type of expectation that defines the perceived quality and thus the user satisfaction (Matzler et al., 1996). Consequently, several contemporary studies have provided method-independent empirical evidence for the assumption of a multi-factorial structure of the user satisfaction construct (see Hölzing (2008) for a discussion of different approaches).

Due to the construct’s importance, literature provides several methods to measure user satisfaction. A cross-sectoral applied approach to measure user expectations and perceptions of service attributes is SERVQUAL (Ladhari, 2009; Parasuraman et al., 1985), which is also applied in the healthcare domain (Akter et al., 2010; Suki et al., 2011). In addition, there are various methods that aim to capture mHealth app users’ perceptions and the resulting evaluation of such apps. For instance, Stoyanov et al. (2015) developed the MARS, a new tool for assessing the quality of mHealth apps. Hereby, the application are as of the MARS range from mindfulness-based apps (Mani et al., 2015) to psychoeducational apps for military members (Jones et al., 2020). de Korte et al. (2018) applied a mixed-method qualitative study based on individual interviews and focus groups, to evaluate a mHealth app in the working context. Finally, Melin et al. (2020) presents the development of a 12-item based questionnaire for assessing user satisfaction with mHealth apps. However, even though the different author teams focus on the evaluation of mHealth apps and the construct user satisfaction, none of the mentioned approaches intend a link of the surveyed user satisfaction to specific features.

Bartikowski and Llosa (2004) provide an analysis of further methods that capture user satisfaction with regard to specific product or service attributes, namely Dual Importance Mapping, Penalty Reward Contrast Analysis, Correspondence Analysis, and the Kano theory of user satisfaction (Kano model). The Kano model which was developed by Kano et al. (1984) has been discussed and applied in several theoretical and empirical research projects (Füller & Matzler, 2008; Löfgren & Witell, 2008). We decided to use the Kano model, since it provides a comprehensive method to analyze the influence of product or service attributes (i.e., features) on user satisfaction.

**Fig. 1:**
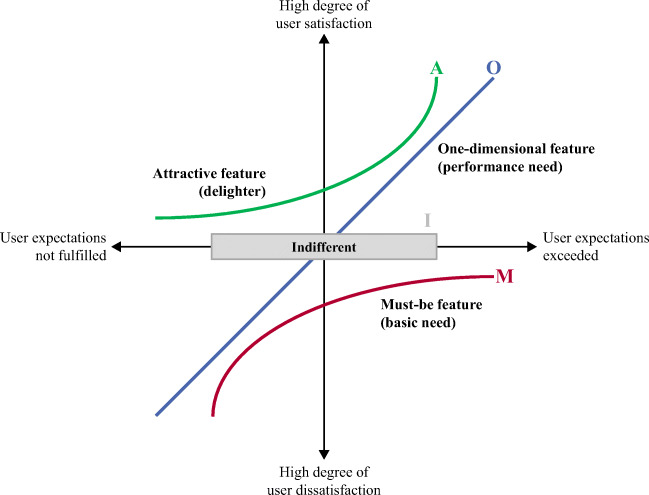
Illustration of the Kano model categories derived from Matzler et al. (1996) and applied to the personal health record context

According to the Kano model, there are four major categories, as listed in Table 1 and illustrated in Fig. 1. These categories depend on actual user expectations and the implementation/nonimplementation of attributes (in our study: features of a PHR) and differ regarding their influence on overall user satisfaction (Berger et al., 1993; Gimpel et al., 2018; Kano et al., 1984; Matzler et al., 1996). The relationship between the performance and importance of attractive and must-be qualities is nonlinear and asymmetric. For instance, some features might perform well but may not be evaluated as very important by users (Matzler et al., 2004).

**Table 1:** List of Kano model categories applied to the personal health record context

Category	User expectations	Effect on user satisfaction	
		if implemented	if not implemented
Attractive quality (delighter)	Users do not expect the implementation of a feature	positive	none
One-dimensional quality (performance need)	Users explicitly demand the implementation of a feature	positive	negative
Must-be quality (basic need)	Users implicitly demand the implementation of a feature	none	negative
Indifferent quality	Users are indifferent to the implementation of a feature	none	none

Furthermore, it is possible to identify the features that have the greatest influence on user satisfaction (Bailom et al., 1996). Thus, the Kano categories lead to a hierarchy of the features that a product (e.g., the PHR app) should contain: providers should fulfill all basic needs, be competitive in terms of performance needs, and offer selected attractive qualities that delight the user to differentiate themselves from competitors, (Berger et al., 1993).

According to Kano (2001), the categories usually follow a specific lifecycle and change over time depending on the experiences or changes in user expectations (from indifferent to attractive to one-dimensional to must-be). New or unknown features should be classified as either indifferent or attractive because users could hardly form distinct expectation levels without substantial usage experience. After gaining more experience, features become part of the user expectations (i.e., one-dimensional) and are eventually recognized as must-be features (Kano, 2001).

### Features of personal health records influencing user satisfaction

Since the late 1990s, PHRs have concerned the research community (e.g., Iakovidis, 1998). They have received increased interest in recent years due to widespread technical capabilities, such as those enabled by smartphones, and their inherent promise to improve health outcomes (Cabitza et al., 2015; Dameff et al., 2019; Wickramasinghe, 2019). The literature has provided various PHR definitions (Roehrs et al., 2017; Tang et al., 2006; Zhou et al., 2019a). At its core, a PHR “can potentially store all the medical records for one patient across multiple health care networks and even countries” (Kao & Liebovitz, 2017, p. 112). The technical implementation can vary considerably, from USB sticks (Kim & Johnson, 2002) and electronic health insurance cards (Pagliari et al., 2007) to web-based portals (Nazi et al., 2010) and smartphone apps (Kharrazi et al., 2012). Within this work, we relate PHRs solely to smartphone apps and follow the definition by Jones et al. (2010):

“[PHRs are] a private, secure application through which an individual may access, manage, and share his or her health information. The PHR can include information that is entered by the consumer and/or data from other sources such as pharmacies, labs, and health care providers.”

Previous PHR research can be grouped into different research streams, inter alia, PHR function evaluation, PHR adoption and attitudes, PHR privacy and security, and PHR architecture (Kaelber et al., 2008). Although Kaelber et al. (2008) emphasized the importance of PHR function evaluation, researchers have primarily focused on PHR adoption and attitudes (Abd-Alrazaq et al., 2019). However, the functions and data elements (i.e., features), are key components of a PHR (Kharrazi et al., 2012). Moreover, PHRs comprise several such features. Within this work, we focus on understanding the PHR feature evaluation.

To identify a comprehensive list of PHR features, we conducted a literature review covering five journals recommended by the Association for Information Systems Special Interest Group Information Technology in Healthcare due to their high relevance in the respective research domain (*Journal of the American Medical Informatics Association*, *International Journal of Medical Informatics*, *Journal of Medical Internet Research*, *Health Systems, and BMC Medical Informatics and Decision Making*). We decided to search specifically for the keywords *PHR Features* and identified 150 publications. Analyzing the titles and abstracts, we narrowed the list to a total of seven publications. Besides, we manually added three publications (Cabitza et al., 2015; Mendiola et al., 2015; Nazi et al., 2010) known to us from our prior research. Extracting the features mentioned in these ten publications resulted in a list of 109 features. Because all these features were derived from detailed feature overviews with large thematic overlaps, we decided not to expand the search string, as the expected knowledge gain would be marginal.

To consolidate the 109 features, we performed an interpretative categorizing analysis using the connecting strategy, which is commonly applied to process healthcare literature (Kerpedzhiev et al., 2019). The connecting strategy is used to identify homogeneous groups of objects and thus is beneficial in the case of several terms with similar meanings (Atkinson, 1992; Maxwell, 2009). Consequently, we merged identical features and pooled features covering similar aspects, and we removed features that were too specific (e.g., Mac-compatible). Subsequently, we refined the feature descriptions in various iterations until the author team reached a consensus.

During this process, it became clear that the feature description of *gamification* by Mendiola et al. (2015) is limited to rewards and does not cover the comparatively new phenomenon in its complexity (Deterding et al., 2011). Therefore, we decided to extend our first literature review by explicitly searching for gamification features in the PHR context. As a result, we manually added three further gamification features (F24 to F26 in Table 2), covering other gamification aspects in PHRs (see Sardi et al., 2017). The resulting 26 PHR features are presented in Table 2.

**Table 2:** Features of Personal Health Record Apps

#	Name and description	References
F1	**Protected personal access.** The app is password protected and requires two-factor authentication (e.g., a code sent to the user’s phone via a text message) for login.	Kharrazi et al. (2012); Kim and Johnson (2002); Maloney and Wright (2010)
F2	**Direct emergency access.** In case of emergency, authorized first aid providers can bypass security features to access medical data (e.g., a user’s current medical condition and history).	Kharrazi et al. (2012); Kim and Johnson (2002); Maloney and Wright (2010)
F3	**Data encryption.** The app stores all data on the phone and servers in encrypted formats.	Halamka et al. (2008)
F4	**Health record.** The app can record personal (e.g., name and insurance number) and medical data (e.g., diagnoses, medications, and immunizations).	Archer et al. (2011); Cabitza et al. (2015); Davis et al. (2017); Dexheimer et al. (2019); Halamka et al. (2008); Kharrazi et al. (2012); Kim and Johnson (2002); Maloney and Wright (2010); Mendiola et al. (2015); Nazi et al. (2010)
F5	**Integration of other health-related records.** The app automatically integrates other health-related records, which allows the user to access his/her complete medical data (e.g., laboratory results, past and current treatments, and medications).	Archer et al. (2011); Cabitza et al. (2015); Davis et al. (2017); Dexheimer et al. (2019); Halamka et al. (2008); Kharrazi et al. (2012); Kim and Johnson (2002); Maloney and Wright (2010); Mendiola et al. (2015); Nazi et al. (2010)
F6	**Integration of trackers.** The user can integrate information from health and physical activity trackers (e.g., Apple Health, Fitbit, and Google Fit) for self-monitoring user-defined indicators (e.g., physical activity, calories, and weight).	Davis et al. (2017); Maloney and Wright (2010); Mendiola et al. (2015)
F7	**Manual upload.** The user can manually upload medical documentation (e.g., test results from private lab facilities), medical reports from specialists (e.g., dentists), and other documents regarding his/her health.	Archer et al. (2011); Cabitza et al. (2015); Davis et al. (2017); Kharrazi et al. (2012); Maloney and Wright (2010)
F8	**Consideration of health predispositions.** The user can import family-related data (e.g., genetic predispositions) from providers of such information (e.g., 23andMe and FamilyTreeDNA).	Archer et al. (2011); Dexheimer et al. (2019); Kharrazi et al. (2012); Nazi et al. (2010)
F9	**Health check/health diary.** The app can regularly query lifestyle-related user data (e.g., smoking and food calories or general wellbeing) and record this information for self-monitoring.	Archer et al. (2011); Dexheimer et al. (2019); Nazi et al. (2010)
F10	**Sharing data with doctors.** The user can authorize doctors to access his/her data (e.g., to get a second opinion, to be referred, or to change to a new family physician more easily).	Cabitza et al. (2015); Davis et al. (2017); Dexheimer et al. (2019); Halamka et al. (2008); Maloney and Wright (2010); Mendiola et al. (2015)
F11	**Sharing data with peers.** The user can share his/her data with relatives and friends (e.g., to ask them for informal advice or to share information that could help them for their own health).	Cabitza et al. (2015); Davis et al. (2017); Dexheimer et al. (2019); Halamka et al. (2008); Maloney and Wright (2010); Mendiola et al. (2015)
F12	**Sharing data with organizations.** The user can authorize his/her insurance and other health-related organizations to access user data (e.g., for bill payment or to speed up reimbursement procedures).	Cabitza et al. (2015); Davis et al. (2017); Dexheimer et al. (2019); Maloney and Wright (2010); Mendiola et al. (2015); Nazi et al. (2010)
F13	**Communication with caregivers.** The app provides an integrated messaging system that enables direct interaction with caregivers (e.g., doctors).	Cabitza et al. (2015); Davis et al. (2017); Halamka et al. (2008); Nazi et al. (2010)
F14	**Community forum.** The app includes a forum that allows the user to ask health-related questions, share experiences, and read responses from other users with similar issues or caregivers.	Davis et al. (2017); Mendiola et al. (2015)
F15	**Social media.** The user can connect the app to social media platforms (e.g., Facebook and Twitter), allowing the user to communicate important health information and events with others.	Davis et al. (2017); Mendiola et al. (2015)
F16	**Health provider registry.** The app provides a searchable health provider registry to let the user know what caregivers and pharmacies are close geographically (e.g., based on geolocation services, such as Google maps).	Kharrazi et al. (2012); Nazi et al. (2010)
F17	**Booking appointments.** The user can book appointments through the app (e.g., ambulatory visits and hospital admissions).	Cabitza et al. (2015); Halamka et al. (2008)
F18	**Reminders.** The app offers automatic reminders and predetermined alerts (e.g., reminders for the ingestion of medicine or upcoming medical appointments).	Cabitza et al. (2015); Davis et al. (2017); Mendiola et al. (2015); Nazi et al. (2010)
F19	**Medication support.** The app offers automated medication support (e.g., by providing guidance regarding drug intolerances and known drug interactions).	Davis et al. (2017); Kharrazi et al. (2012); Mendiola et al. (2015); Nazi et al. (2010)
F20	**Care plan.** The app can provide the user with individual plans of action for reaching target goals, including specific, executable steps to guide the process (e.g., personal aftercare plan after a hospital stay).	Davis et al. (2017); Mendiola et al. (2015)
F21	**General education.** The app provides basic educational material about a disease or condition, including prevention through vaccines, causes, treatment, or management.	Davis et al. (2017); Mendiola et al. (2015); Nazi et al. (2010)
F22	**Virtual assistant.** The app includes a virtual assistant (e.g., an artificial intelligence-based chatbot), which provides personalized health information and guidance regarding preventive health recommendations and symptom analysis.	Archer et al. (2011); Davis et al. (2017); Dexheimer et al. (2019); Maloney and Wright (2010); Mendiola et al. (2015)
F23	**Health rewards.** The app rewards the user with points and badges as health objectives are achieved (e.g., for the undergoing of annual dental prophylaxis).	Mendiola et al. (2015)
F24	**Motivational messages.** The app provides motivational messages (e.g., about the importance of preventive medical checkups) to seek needed care.	Hors-Fraile et al. (2018); Kerns et al. (2013)
F25	**Challenges and quests.** The app provides health-related challenges and quests (e.g., to engage participation and thus address health topics more), which take place among users in a collaborative or single mode.	AlMarshedi et al. (2015); Hutchison et al. (2014); Lister et al. (2014); Miller et al. (2016)
F26	**Personalized avatars.** The app provides personalized avatars that represent the user and his/her current health status (e.g., to help the user visualize and better take charge of their health).	Borghese et al. (2013); Lentelink et al. (2013); Miloff et al. (2015)

Because the 26 features in this study cover various aspects of PHRs and because we further expect significant differences between potential users in Germany and Denmark, we hypothesize the following:

1. The effect of PHR features on the satisfaction of potential users follows a multi-categorial structure with features being categorized as basic needs (M), performance needs (O), delighters (A), indifferent (I), or reverse (R).

### User characteristics influencing personal health record feature evaluation

**Fig. 2:**
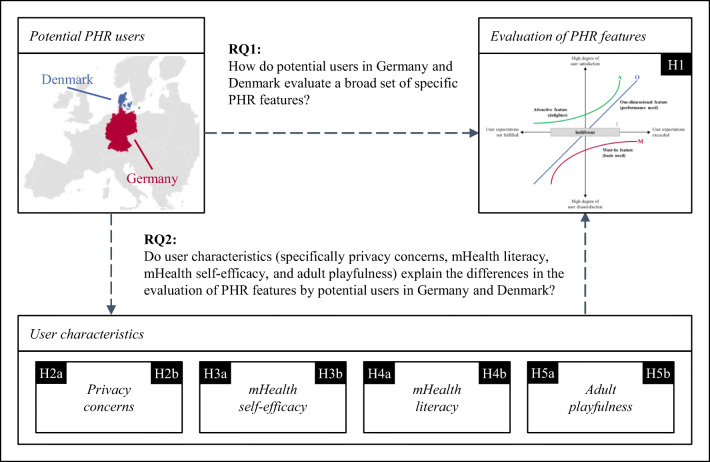
Research model

Figure 2 displays the research model and hypotheses addressing the two research questions of this study. Next, we introduce the four user characteristics and hypothesize their influence on the PHR feature evaluation.

#### Privacy concerns

*Privacy* typically connotes something positive (Warren & Laslett, 1977) that must be protected or preserved (Margulis, 2003). This especially holds for personal medical data in a digitalized world, as it is particularly sensitive and exposed to privacy incidents (Anderson, 2007; Appari & Johnson, 2010). Numerous publications have dealt with the role of privacy in digital health (e.g., Anderson & Agarwal, 2011; Angst & Agarwal, 2009; Winston et al., 2016).

Because privacy is a latent construct and thus cannot be measured directly, research often employs the concept of *privacy concerns* as a proxy for privacy (Li, 2011; Smith et al., 1996; Smith et al., 2011). Privacy concerns are “the extent to which individuals are disturbed about the information collection practices of others [e.g., organizations] and how the acquired information will be used” (Angst & Agarwal, 2009, p. 342). Several studies have shown that Germans have higher privacy concerns than citizens in most other countries (e.g., Bellman et al., 2004; IBM, 1999; Miller, 2017). Most authors attribute this to German’s historical legacy: in the twentieth century, two regimes in Germany heavily surveilled their citizens to retain power (Whitman, 2004). Privacy concerns have become deeply engraved in the Germans’ collective memory (Flaherty, 2014). Accordingly, we pose the following hypothesis:

- **Hypothesis 2a:** Germans tend to have higher privacy concerns than Danes.

In healthcare digitalization, privacy concerns are one of the major barriers for individuals to accept and use healthcare technologies (Anderson, 2007). This applies especially to PHRs because they constitute a new way that personal health data are stored, shared, and processed by the multiple parties involved in the healthcare system (Li & Slee, 2014). Furthermore, previous research has suggested that safeguarding privacy increases individuals’ satisfaction (e.g., George & Kumar, 2014; Khalaf Ahmad & Ali Al-Zu’bi, 2011; Nayeri & Aghajani, 2010). Because several PHR features are privacy-related (e.g., F1 or F3 in Table 2), require sensitive personal medical data (e.g., F8 or F19), or involve interfaces with other services (e.g., F6 or F12 in Table 2), we argue that privacy concerns affect user satisfaction regarding PHR features. Thus, we hypothesize the following:

- **Hypothesis 2b:** Privacy concerns influence the evaluation of some PHR features.

#### mHealth literacy

Researchers have a growing interest in *mHealth literacy* due to the increasing use and acceptance of smartphones in health care (Birkhoff & Moriarty, 2020; Lin & Bautista, 2017; Messner et al., 2019). Although thematic overlaps exist between health literacy, eHealth literacy, and the comparatively new construct of mHealth literacy, researchers have argued that the constructs should be distinguished (Ahmed, 2017; Lin & Bautista, 2017; van der Vaart & Drossaert, 2017). Following Lin and Bautista (2017), we define mHealth literacy as “the ability to use mobile devices to search, find, understand, appraise, and apply health information to address or solve a health problem” (p. 347).

Individuals mHealth literacy is context-dependent (Ćwiklicki et al., 2020; Messner et al., 2019) and can vary across countries (Okan et al., 2019). Researchers often underline the high digitalization level of health care in Denmark (e.g., Bertelsmann Stiftung, 2018; Kierkegaard, 2013) and the slow adoption of digital healthcare solutions in Germany (Nohl-Deryk et al., 2018). The overall level of mHealth literacy must align with digitalization because being literate about mHealth apps is one prerequisite for using them adequately (Kreps, 2017). Therefore, in line with previous research results (European Commission, 2014), we argue that Danes have a higher level of mHealth literacy than Germans. Conversely, we posit the following:

- **Hypothesis 3a:** Germans tend to have lower mHealth literacy than Danes.

Inadequate literacy in health care (e.g., insufficient self-management skills and limited medication adherence) is associated with lower patient satisfaction (Altin & Stock, 2016; MacLeod et al., 2017). In contrast, Zhang et al. (2018) found that mHealth literacy significantly increases the satisfaction of mHealth apps users and attributes this relation to a better match of user expectations and experience. Most PHR features require a certain level of mHealth literacy to provide added value to users (e.g., F9, F18 in Table 2). Hence, a higher level of mHealth literacy may also lead to a higher level of user satisfaction and, thus, to a different evaluation of some of the PHR features. We pose the following hypothesis:

- **Hypothesis 3b:** mHealth literacy influences the evaluation of some PHR features.

#### mHealth self-efficacy

*Self-efficacy* refers to individuals’ confidence or belief in their ability to complete a task (Bandura, 1986). Furthermore, self-efficacy has a well-established, positive influence on the health status and health behavior of individuals of all ages (Grembowski et al., 1993). We follow Fox and Connolly (2018) and define mHealth self-efficacy as the “individuals’ perceived ability to use m-health to manage their health” (p. 999).

Contrary to *literacy*, the efficacy judgment can over- or underestimate true ability. Thus, although self-efficacy usually correlates with literacy, it does not necessarily reflect actual literacy (Cheema & Skultety, 2017). Previous research has reported significant positive correlations between mHealth literacy and self-efficacy (e.g., Berens et al., 2018). Based on the close link between literacy and self-efficacy and based on prior work that found a lower level of mHealth literacy for Germans compared to Danes (European Commission, 2014), we hypothesize the following:

- **Hypothesis 4a:** Germans tend to have a lower mHealth self-efficacy than Danes.

Furthermore, empirical studies suggest a significant positive relationship between self-efficacy and satisfaction because self-efficacy improves task performance and increases users’ perceived service value (e.g., Machmud, 2018; McKee et al., 2006). We assume that this relation also applies to mHealth self-efficacy and mHealth user satisfaction. Our list of PHR features contains several features (e.g., F7, F13, F17 in Table 2) for which users should demonstrate a certain level of mHealth self-efficacy to use them effectively. Accordingly, we posit the following hypothesis:

- **Hypothesis 4b:** mHealth self-efficacy influences the evaluation of some PHR features.

#### Adult playfulness

Using gamification in mHealth apps is a relatively young and emerging trend (Schmidt-Kraepelin et al., 2020) that has the potential to promote behavioral health changes (Miller et al., 2016), to improve user self-management (Charlier et al., 2016), and to overcome a loss of interest and user engagement over time (Schmidt-Kraepelin et al., 2020). Several contemporary studies have applied various “game design elements in non-game contexts” (Deterding et al., 2011, p. 10), for example, in chronic disease rehabilitation (AlMarshedi et al., 2015) and mental health (Miloff et al., 2015). By analyzing 143 apps from the Apple App Store and the Google Play Store, Schmidt-Kraepelin et al. (2020) identify eight archetypes of gamification that are applied in mHealth apps (e.g., competition and collaboration, episodical compliance tracking, internal rewards for self-set goals). Previous research has shown that gamification can increase user satisfaction by fulfilling psychological needs, such as social relatedness (Sailer et al., 2017) and by increasing motivation or improving users’ emotional experiences (Sardi et al., 2017).

Researchers frequently use *adult playfulness* to measure individuals’ receptiveness to gamification elements (e.g., Codish & Ravid, 2015; Müller-Stewens et al., 2017; Poncin et al., 2017). According to Glynn and Webster (1992), adult playfulness is “an individual trait, a propensity to define (or redefine) an activity in an imaginative, nonserious or metaphoric manner so as to enhance intrinsic enjoyment, involvement, and satisfaction” (p. 85).

In the only available cross-country study on adult playfulness, Pang and Proyer (2018) concluded that societal rules and cultural factors might affect playfulness in a society. Anecdotal evidence suggests the Danish culture is more liberal and progressive than many other cultures, including the German culture (Allen, 2012; Hoefler & Vejlgaard, 2011; Jensen, 2017). Cultural surveys reflect these libertarian values with comparably low values of power distance and high values of gender egalitarianism for Denmark and other Scandinavian countries (Hofstede Insights, 2020; House et al., 2011). Libertarian values may go along with higher playfulness among adults because liberal and progressive settings encourage play to a greater extent than conservative settings. Hence, despite limited prior evidence, we pose the following hypothesis:

- **Hypothesis 5a:** Germans tend to have a lower adult playfulness than Danes.

Adult playfulness may influence the evaluation of PHR features. For example, our list of PHR features contains several gamification elements that can fulfill social relatedness (e.g., F14, F15) or increase user motivation (e.g., F24, F25 in Table 2). Gamification elements in mHealth apps may appeal more to those with higher adult playfulness and less to those with lower adult playfulness leading them to have greater preferences for these features. To conclude, we propose the following:

- **Hypothesis 5b:** Adult playfulness influences the evaluation of some PHR features.

## Research method

To address our research objective of evaluating PHR features by potential users from Germany and Denmark, we decided to use the Kano method,^3^ due to its ability to account for individual preferences regarding each PHR feature. We operationalized the four user characteristics (Fig. 2) as factors based on the existing literature and conducted an online survey to test the theoretical hypotheses.

### Kano method

**Fig. 3:**
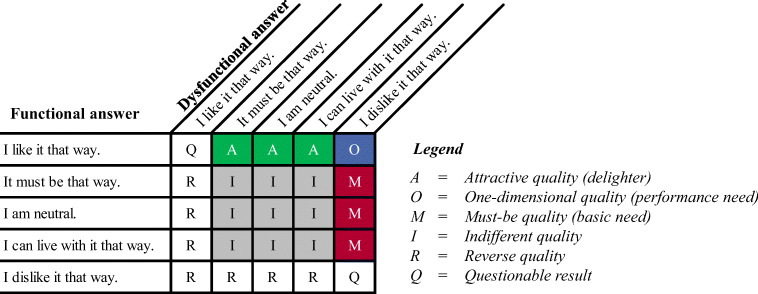
Evaluation scheme for the derivation of Kano categories

The PHR features are classified depending on the users’ answers to both a functional and a dysfunctional question (Berger et al., 1993; Gimpel et al., 2018; Kano et al., 1984; Matzler et al., 1996). The functional question refers to the user’s reaction if the respective feature is present, whereas the dysfunctional question refers to the reaction if the feature is not present. Each question has five possible answers (Fig. 3). The combination of answers to these question pairs can be interpreted individually for each feature and leads to a specific category, as illustrated in Fig. 3. Hereby, the evaluation scheme is not appropriate to draw conclusions about the importance of individual features (see Lee & Newcomb (1997) for the design of an importance matrix based on the Kano questionnaire).

The most intuitive and easiest way to determine the resulting Kano model categorization of an attribute is the mode (Berger et al., 1993). However, solely using the mode leads to a lack of further information about other frequently appearing categorizations, especially if the shares of categories are of similar size (Schaule, 2014). Thus, further analyses are common and necessary to determine the categorization significance (Gimpel et al., 2018; Schaule, 2014).

Lee and Newcomb (1997) developed the variable *category strength*, which can be used to determine whether an attribute belongs to only one category. The category strength is calculated as the difference between the shares of the most and second-most frequently assigned categories. It may be considered statistically significant if it is equal to or greater than 6%; otherwise, the attribute belongs to a mixed category (Lee & Newcomb, 1997). The approach proposed by Fong (1996) supports a categorization if the category strength is higher than a calculated reference value that is based on the observed categorization frequencies and the overall sample size. If the categorization based on the mode is not supported by Fong’s approach, Berger et al. (1993) proposed applying the (A, O, M) < > (I, R, Q) rule, where the categorizations are divided into two groups based on their (non)influence on user satisfaction. A categorization of A (attractive), O (one-dimensional), or M (must-be) means that an attribute influences user satisfaction. In contrast, a categorization of I (indifferent), R (reverse), or Q (questionable) indicates that an attribute has no (positive) influence on user satisfaction. The proposed evaluation rule is applicable if both the most and second-most categorizations belong to different groups (e.g., A and I). Given the latter, the rule is executed by first determining the group with the highest share of categorizations of the overall sample and then selecting the most frequently chosen category within this group.

In the current work, we proceed in the same way as Gimpel et al. (2018) to determine the resulting categories of the features. Therefore, we assign categories to the features based on the mode if the *category strength* is significant at a 10% level, according to Fong’s approach. If the respective *category strength* is not significant and the (A, O, M) < > (I, R, Q) rule is applicable, we execute this rule. If the (A, O, M) < > (I, R, Q) rule is not applicable, we assign the feature to a mixed category. In this case, we also name all categories that do not significantly differ according to Fong’s approach compared to the most frequently chosen category.

### Operationalization of user characteristics

We derived all four user characteristics (Fig. 2) based on the existing literature and operationalized them on a seven-point Likert scale (1 = strongly disagree to 7 = strongly agree). The respective measures are provided in Appendix 1.

To measure *privacy concerns* regarding personal health data, we used the 15-item scale from Angst and Agarwal (2009). Angst and Agarwal (2009) adapted one of the most influential scales to measure individuals’ concerns for information privacy, originally developed and tested by Smith et al. (1996).

To operationalize *mHealth literacy*, we followed the approach by Lin and Bautista (2017). They used the widely adopted and comprehensively tested eight-item scale developed by Norman and Skinner (2006) and replaced the word *computer* with *mobile phone*. Lin and Bautista (2017) suggested that mHealth literacy is a higher-order construct including two mHealth factors: information searching (four items) and information appraisal (four items). Information searching comprises the skill to search for and find health-related information on a smartphone. In contrast, information appraisal covers the capability to understand, appraise, and apply health-related information on a smartphone. Given the inconsistency of the underlying factor structure across previous studies (Juvalta et al., 2020), we decided to test both operationalizations (single-factor and two-factor structures) and report the two-factor results.

For mHealth self-efficacy, we used the three-item scale from Fox and Connolly (2018). Fox and Connolly built on the work by Kim and Park (2012) on a measurement instrument consisting of six items.

We followed Proyer (2012) for adult playfulness and used the Short Measure of Adult Playfulness (SMAP). The SMAP consists of five items and is based on the need for a play scale (Jackson, 1974), the Adult Playfulness Scale (Glynn & Webster, 1992), and a list of playfulness qualities by Barnett (2007).

### Survey

Before conducting the survey, four fellow researchers and six other voluntary participants pretested the English survey. Based on their feedback, we added further explanations and examples to the features’ descriptions and divided the survey into three mandatory parts and one optional part.

**Fig. 4:**
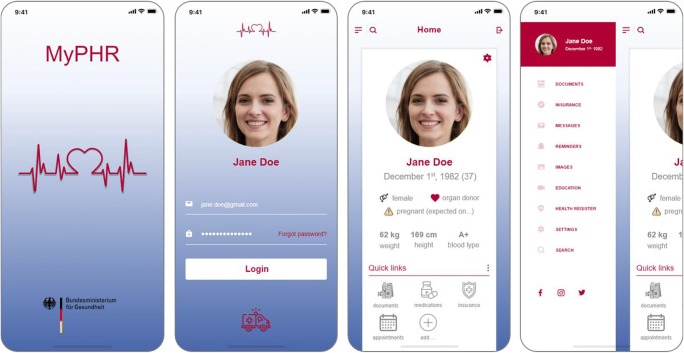
Exemplary screenshots of a fictional PHR

In the first part, we presented screenshots of a fictional PHR app to give participants a basic impression of the potential PHR app. We put them into the situation of evaluating its features, similar to an app store site (see Fig. 4 in Appendix 2). In the second part, participants were asked one functional and one dysfunctional question for each of the 26 features. For example, for Feature F13 (Table 2), the functional question was as follows: “Communication with caregivers. The app provides an integrated messaging system that enables direct interaction with caregivers (e.g., doctors).” The dysfunctional question was as follows: “‘Communication with caregivers’ is not provided.” The third part contained the scales for privacy concerns, mHealth literacy, mHealth self-efficacy, adult playfulness and the demographic data (gender, age, level of education, employment status, usage of healthcare-related apps, and understanding of the survey).

The optional part contained questions about the culturally influenced values and sentiments of the participants. We used this part to support the cultural representativeness of the sample regarding Germany and Denmark. As a measure, we used the Values Survey Module questions covering the six Hofstede dimensions (Hofstede et al., 2010).

The survey ran from February through March 2020. We recruited participants via social media and email and incentivized them through a lottery of vouchers for an online retailer. Overall, 323 participants from 27 different countries completed the survey. Given the focus on Denmark and Germany, we excluded 45 valid responses from other countries. Furthermore, we excluded six participants because they sped through the survey or stated difficulties in understanding the survey questions.

The final sample comprises 274 participants, including 215 Germans and 59 Danes. Both men (52%) and women (48%) completed the survey. The sample mostly consists of students (51%) and employees (46%). The age of participants was between 18 and 73 years (average age 28.9 years). Most participants (84%) indicated having at least a university degree). The majority of participants reported never using healthcare-related apps (45%) or using them less than once a month (27%). Table 11 (Appendix 3) describes the composition of participants in both countries. Although the sample characteristics are similar in several parts, there may be a risk of bias due to the comparatively unbalanced sample size (Guyatt et al., 2011).

Out of the final sample, 208 participants (76%) completed the optional part, including 157 Germans and 51 Danes. Our assessment of the Hofstede dimensions (Table 12, Appendix 3) reveals that the subsamples’ cultural differences are qualitatively comparable with the differences between the original Hofstede values for Germany and Denmark (Hofstede Insights, 2020), indicating the cultural representativeness of the sample.

## Results

This section first presents the overall evaluation of the 26 PHR features between Germans and Danes before testing the hypothesized differences in the user characteristics (H2a to H5a) and their influence on the feature evaluation (H2b to H5b).

### Evaluation of personal health record features

Table 3 presents the categorization of PHR features according to the Kano model, split into the German and Danish subsamples. For both subsamples, we present the category strength and final categorization of each feature. The results indicate that the categorization of delighters (attractive quality) was assigned most frequently in both subsamples (Germany: 11; Denmark: 14), whereas the categorization of performance needs (one-dimensional quality) is very rare (Germany: 0; Denmark: 1). Furthermore, *protected personal access* (F1) and *data encryption* (F3) are considered by both Germans and Danes to be basic needs (must-be quality). Thus, the implementation of these security features is not rewarded, but downside risks exist if they are not implemented. Consequently, these two features should be implemented during the development of the PHR. This result is not unexpected, since data protection and high security standards are important issues regarding mobile applications in general (Jain & Shanbhag, 2012). This applies in particular to personal health data, which is among the most sensitive personal data (Martínez-Pérez et al., 2015; Müthing et al., 2019; Zhou et al., 2019b). However, it should be emphasized that the resulting evaluation is neither a question of the clinical necessity of these two features, nor dependent of the type of technical implementation. The categorization as must-be qualities is solely based on the contribution of these two features to the personal satisfaction of potential users in Germany and Denmark. The survey participants categorized several features as indifferent (Germany: 10; Denmark: 4). Also, *social media* (F15) is considered to have a reverse quality in Germany, whereas Danes categorized no feature as having a negative effect on user satisfaction. Finally, for a few features, the categorization was not significant, and the features were assigned a mixed category (Germany: 2; Denmark: 5).

**Table 3:** Empirical results of the personal health record feature evaluation via the Kano model

#	Short description	Germany (*n* = 215)		Denmark (*n* = 59)		Diff.
		Category strength	Category	Category strength	Category	
F1	Protected personal access	13% *	M	63% *	M	no
F2	Direct emergency access	5% ^1^	A	2% ^2^	Mixed (A, O)	yes
F3	Data encryption	20% *	M	69% *	M	no
F4	Health record	8% *	I	20% *	O	yes
F5	Integration of other health-related records	5% ^1^	A	8% ^2^	Mixed (O, A)	yes
F6	Integration of trackers	2% ^1^	A	47% *	A	no
F7	Manual upload	7% ^1^	A	3% ^2^	Mixed (A, O)	yes
F8	Consideration of health predispositions	24% *	I	27% *	A	yes
F9	Health check/health diary	22% *	I	47% *	A	yes
F10	Sharing data with doctors	8% *	A	2% ^2^	Mixed (A, O)	yes
F11	Sharing data with peers	3% ^2^	Mixed (I, R)	17% *	I	yes
F12	Sharing data with organizations	4% ^2^	Mixed (R, I)	36% *	A	yes
F13	Communication with caregivers	8% *	I	58% *	A	yes
F14	Community forum	15% *	I	36% *	A	yes
F15	Social media	56% *	R	2% ^2^	Mixed (R, I)	yes
F16	Health provider registry	22% *	A	64% *	A	no
F17	Booking appointments	29% *	A	63% *	A	no
F18	Reminders	10% *	A	68% *	A	no
F19	Medication support	5% ^1^	A	53% *	A	no
F20	Care plan	8% *	A	47% *	A	no
F21	General education	11% *	A	49% *	A	no
F22	Virtual assistant	14% *	I	47% *	A	yes
F23	Health rewards	14% *	I	24% *	A	yes
F24	Motivational messages	19% *	I	3% ^1^	I	no
F25	Challenges and quests	16% *	I	3% ^1^	I	no
F26	Personalized avatars	30% *	I	2% ^1^	I	no

Legend: * = Categorization according to Fong’sapproach^1^ = (O + A + M) < > (I + R + Q) rule applicable^2^ = (O + A + M) < > (I + R + Q) rule not applicable A = Attractive quality (delighter).

O = One-dimensional quality (performance need)M = Must-be quality (basic need)I = Indifferent qualityR = Reverse quality

Overall, 14 measures (54%) exhibit different categorizations between Germans and Danes. For five of these features, the categorization in one of the subsamples corresponds to the most frequent result of the mixed category categorization in the other subsample (F2, F7, F10, F11, and F15 in Table 2). Although these categorizations are not equal, the tendencies are more similar. We notice clear differences between Germany and Denmark for nine of the features. Most of these differences follow one of the two following patterns. First, features that are categorized as indifferent by Germans are frequently categorized as one-dimensional qualities or delighters by Danes (F4, F8, F9, F13, F14, F22, and F23 in Table 2). Second, in some cases, features are categorized as delighters in Germany, whereas Danes categorized them ambiguously as performance needs and delighters (F2, F5, F7, and F10 in Table 2). The feature *sharing data with organizations* (F12) stands out in that most Germans categorized it as a reverse quality. Not only do they not want the feature, but they also do not expect this feature to be there, whereas Danes categorized the feature as a delighter.

We underline these results by examining the feature categorization in more detail on the participant level. For both Germans and Danes, Table 4 presents the minimum, mean, and maximum number of feature categorizations and the standard deviation per survey participant. Furthermore, Table 4 lists the share of participants who categorized none or at least nine (i.e., more than one-third) of the features as a specific Kano model category.

**Table 4:** Statistics regarding the number of Kano categories by survey participants

	Germans (*n* = 215)						Danes (*n* = 59)					
	Min^a^	Mean^a^	Max^a^	Sd^a^	None^b^	≥ 9^b^	Min^a^	Mean^a^	Max^a^	Sd^a^	None^b^	≥ 9^b^
Attractive quality	0	7.04	20	4.63	10%	37%	1	13.00	21	5.58	0%	81%
One-dimensional quality	0	3.17	17	3.18	17%	7%	0	3.03	11	2.32	3%	3%
Must-be quality	0	2.08	8	1.84	23%	0%	0	2.53	7	1.58	10%	0%
Indifferent quality	0	9.27	25	4.93	1%	50%	0	5.90	21	4.85	8%	20%
Reverse quality	0	4.40	24	3.98	11%	13%	0	1.54	10	2.27	44%	2%
Questionable result	0	0.06	3	0.30	96%	0%	0	0.00	0	0.00	100%	0%

^a ^reference value: number of features; ^b^ reference value: number of survey participants

Overall, the data support hypothesis H1 for both the German and Danish subsamples. However, we also see clear differences between the German and Danish subsamples. The features with indifferent quality are dominant for German participants: every other German (50%) categorized at least 9 of the 26 features as having indifferent quality. In Denmark, this is only 1 in 5 (20%). Further, 81% of all Danish participants categorized at least nine features as delighters, compared to only 37% of Germans. The low proportion of questionable results in both subsamples indicates good data quality. In summary, several differences in the evaluation of features in Germany and Denmark were found, which we aim to explain in the next part based on certain user characteristics.

### Explanatory power of user characteristics

We first evaluate the psychometric adequacy of the measurement model for user characteristics before we test the research hypotheses.

#### Measurement model assessment

To evaluate the psychometric adequacy, we conducted an exploratory factor analysis (EFA) with oblique rotation (reported in Table 9 in Appendix 1). To assess the suitability of the sample data for the factor analysis, we calculated the Kaiser-Meyer-Olkin (KMO) measure of sampling adequacy (Kaiser, 1970) and Bartlett’s test of sphericity (Bartlett, 1950). Both results (KMO: .83; Bartlett’s test of sphericity: *p* < .001) indicated good prerequisites for the EFA. Via Horn’s parallel analysis and assessment of interpretability, we determined the number of factors to extract as eight (Horn, 1965). Tabachnick and Fidell (2013, p. 651) suggested using oblique rotation when a high overlap exists in the variance (≥ 10%) of some oblique rotated factors.

Correlations that exceed the associated factor correlation threshold of .32 (Table 5) were in line with the theoretical conceptualization and well-established in the literature. First, we anticipated a strong link between all four first-order concerns for information privacy constructs (*collection*, *errors*, *unauthorized access*, and *secondary use*), as they are often aggregated into an overall score (Smith et al., 1996; Stewart & Segars, 2002). Second, we expected a strong correlation between the two factors of mHealth literacy (*mHealth information seeking* and *mHealth information appraisal*) because these factors are grounded in a single construct (Norman & Skinner, 2006).

**Table 5:** Construct Correlations and Distributions

Construct	Mean	SD	Alpha	No. of items	1	2	3	4	5	6	7	8
1. Collection (privacy concerns)	3.99	1.55	0.90	4	**0.87**							
2. Errors (privacy concerns)	5.04	1.13	0.87	4	0.35***	**0.86**						
3. Unauthorized access (privacy concerns)	6.02	1.06	0.88	3	0.36***	0.65***	**0.90**					
4. Secondary use (privacy concerns)	6.34	0.89	0.80	4	0.36***	0.42***	0.71***	**0.80**				
5. mHealth information searching (mHealth literacy)	5.24	1.20	0.89	4	0.03	0.00	−0.01	−0.06	**0.87**			
6. mHealth information appraisal (mHealth literacy)	4.85	1.40	0.86	3	0.05	0.01	−0.01	−0.11	0.73***	**0.88**		
7. mHealth self-efficacy	5.62	1.27	0.87	3	−0.02	−0.02	−0.08	−0.06	0.21***	0.30***	**0.89**	
8. Adult playfulness	4.83	1.26	0.87	5	−0.01	−0.09	−0.12*	−0.04	0.12*	0.18**	0.18**	**0.82**

* *p* < .05; ** *p* < .01; *** *p* < .001; bold diagonals represent the square root of the average variance extracted for multi-item scales; product term is standardized; *N* = 274

Means, standard deviations, scale alphas, and inter-construct correlations are summarized in Table 5. Cronbach’s alpha (≥ 0.80) suggests that all scales have convergent validity (Cronbach, 1951). Discriminant validity was confirmed using two assessments. First, indicators should load stronger on their corresponding construct than on other constructs in the model (Gefen & Straub, 2005). Further, items with factor loadings above .55 can be considered good (Comrey & Lee, 2016) and cross-loadings below .32 are negligible (Tabachnick & Fidell, 2013, p. 654). While all items loaded stronger on their corresponding construct and had good factor loading, one item (HIA1) had a cross-loading above the threshold of .32 and was dropped. Second, the square root of the average variance extracted (bold diagonal in Table 5) should be larger than the inter-construct correlations (Fornell & Larcker, 1981). Because both criteria were met, we conclude that the items and constructs exhibit adequate discriminant validity. Finally, we conducted a confirmatory factor analysis to evaluate the model fit of the eight-factor solution. Following the guidelines by Jackson et al. (2009), we calculated several fit measures (see Table 10 in Appendix 1). The fit measures indicate a good model fit and support the eight-factor solution, initially derived by the EFA.

#### Influences on feature evaluation

To test the hypotheses, we first test whether significant differences exist in the user characteristics between Germans and Danes (H2a to H5a). Then we identify potential influences of the user characteristics on the evaluation of PHR features (H2b to H5b). For the first step, we applied the one-tailed Welch’s *t*-test and the one-tailed Mann–Whitney U-test on the factor and sub-factor scores of the user characteristics. The means, standard deviations, and test results are summarized in Table 6.

**Table 6:** Differences between Germany and Denmark regarding the potential influencing user characteristics

User characteristics	Germany		Mean comp.	Denmark		*t*-value	*W*-value	Hypothesis
	Mean	SD		Mean	SD			
Privacy concerns^a^	5.44	0.77	≥	4.92	0.92	−3.83 ***	4338 ***	H2a: Supported
Collection	3.96	1.53	≥	3.60	1.38	−2.01 *	5342 *	
Errors	5.15	1.12	≥	4.56	1.06	−3.81 ***	4342 ***	
Unauthorized access	6.21	0.84	≥	5.42	1.30	−3.88 ***	4194 ***	
Secondary use	6.42	0.79	≥	6.10	1.16	−1.99 *	5747	
mHealth literacy^b^	5.00	1.17	≤	5.43	1.01	2.54 **	7436 *	H3a: Supported
mHealth information searching	5.22	1.19	≤	5.52	1.09	1.34	6999	
mHealth information appraisal	4.77	1.42	≤	5.33	1.24	2.99 **	7675 **	
mHealth self-efficacy	5.67	1.19	≤	6.18	0.85	3.96 ***	8126 ***	H4a: Supported
Adult playfulness	4.72	1.20	≤	5.24	1.43	2.29 *	7794 **	H5a: Supported

* *p* < .05; ** *p* < .01; *** *p* < .001; ^a^ average of the four first-order construct scores of collection, errors, unauthorized access, and secondary use; ^b^ average of the two first-order construct scores of mHealth information searching and mHealth information appraisal

The data reveal significant factor-level differences between Germany and Denmark for all four user characteristics and, therefore, support the hypotheses (H2a to H5a). According to the data, Germans have significantly higher privacy concerns, lower mHealth literacy, lower mHealth self-efficacy, and lower adult playfulness than Danes.

To test the user characteristics’ influences on the evaluation of PHR features (H2b to H5b), we followed a three-step approach. First, we subdivided the sample for each of the four user characteristics in three groups using a quartile-based sample-split approach. The first group (*low*) consists of participants that scored in the lower quartile of the respective characteristic. The second group (*middle*) includes participants from both the second and third quartiles jointly. The third group (*high*) comprises participants from the upper quartile of the respective variable. Second, we applied the Kano model to each subsample 12 times. Because the second research question focuses on differences in the PHR feature evaluation, we focus on these 14 features with ascertained differences between Germany and Denmark (see column Diff. in Table 3). Table 7 displays the different results regarding the feature *consideration of health predispositions* (F8) and the user characteristic *privacy concerns*. The table lists the relative share of chosen categories, category strength, and final categorization of the feature. Thus, this approach is appropriate for identifying evaluation differences between the different groups.

**Table 7:** Exemplary categorization of *consideration of health predispositions* (F8) for low, middle, and high privacy concerns

Quartile (*group*)	A	O	M	I	R	Q	Category strength	Category
1st (*low*)	46%	4%	3%	38%	8%	0%	8% ^1^	A
2nd and 3rd (*middle*)	31%	7%	1%	49%	10%	1%	18% *	I
4th (*high*)	22%	1%	6%	49%	21%	0%	27% *	I

Legend: * = Categorization according to Fong’s approach

^                  1^ = (O + A + M) < > (I + R + Q) rule applicable

A = Attractive quality (delighter)

O = One-dimensional quality (performance need)

M = Must-be quality (basic need)

I = Indifferent quality

R = Reverse quality

Q = Questionable result

The complete categorization results of the 14 PHR features for the three groups and all four user characteristics are provided in Table 13 (Appendix 4). Third, we compare the results for the low and high quartiles from the second step (Table 13, Appendix 4) with the categorizations of Germans and Danes (Table 3) to explore similarities that can explain the categorization of a feature. To identify potential explanations, we use the grammar of the formal language theory (Harrison, 1978). This formalization assigns a mathematical meaning to the categorizations, which is useful for automated relationship verification. The following relationships apply:

$$ {\displaystyle \begin{array}{c}\left[\left\{{x}_i^{Germany}\right\}\circ \left\{{x}_i^{Denmark}\right\}=\left\{{y}_i^{j, low}\right\}\circ \left\{{y}_i^{j, high}\right\}\right]\bigwedge \left[{\overline{z}}^{j, Germany}<{\overline{z}}^{j, Denmark}\right]\longrightarrow potential\ explanation\\ {}\left[\left\{{x}_i^{Denmark}\right\}\circ \left\{{x}_i^{Germany}\right\}=\left\{{y}_i^{j, low}\right\}\circ \left\{{y}_i^{j, high}\right\}\right]\bigwedge \left[{\overline{z}}^{j, Germany}>{\overline{z}}^{j, Denmark}\right]\longrightarrow potential\ explanation\end{array}} $$

where

*x*categorization of feature *i* in the respective country

*y*categorization of feature *i* in the respective subsample of user characteristic *j*𝑗

$\overline{z}$the arithmetic mean of user characteristic *j* in the respective country

*x*, *y* ∈{*A*, *O*, *M*, *I*, *R*, *Q*, *Mixed* ()}

z ∈ ℝ^+^

*i* ∈ {2,4,5,7,8,9,10,11,12,13,14,15,22,23}

*j ∈*{*privacy concerns, mHealth literacy, mHealth self-efficacy, adult playfulness*}

Table 8 presents the results. Potential identified explanations are labeled with ✓. Furthermore, identified similarities based on comparisons between mixed categories (e.g., {*A*}{*Mixed*(*O*, *A*)} ≈ {*A*}{*Mixed*(*O*, *A*, *I*}) are labeled with (✓). A match is assumed if the first two categorizations between the mixed categories match. The following example refers to the feature *consideration of health predispositions* (F8) and illustrates the comparison procedure. According to Table 3, Germans evaluated F8 as an indifferent quality, whereas Danes evaluated F8 as a delighter. According to Table 13 (Appendix 4), participants with low privacy concerns evaluated F8 as a delighter, whereas participants with high privacy concerns evaluated F8 as an indifferent quality. According to Table 6, the arithmetic mean of privacy concerns in Germany (5.44) is higher than in Denmark (4.92). Applying the algorithm results in [{*A*}{*I*} = {*A*}{*I*}] ⋀ [5.44 > 4.92]. Thus, the comparison indicates a potential reason Germans evaluate F8 as indifferent and why Danes evaluated it as a delighter: specifically, because Germans are more privacy-sensitive while Danes are less privacy-sensitive.

**Table 8:** Potential influences of user characteristics on the evaluation of features in Germany and Denmark

#	Feature	Germany (*n* = 215)		Denmark (*n* = 59)			Potential explanatory user characteristics			
		Category strength	Category	Categorystrength	Category		Privacy Concerns	mHealth Literacy	mHealth Self-Efficacy	Adult Playfulness
F2	Direct emergency access	5% ^1^	A	2% ^2^	Mixed (A, O)				✓	(✓)
F4	Health record	8% *	I	20% *	O					
F5	Integration of other health-related records	5% ^1^	A	8% ^2^	Mixed (O, A)				(✓)	
F7	Manual upload	7% ^1^	A	3% ^2^	Mixed (A, O)				✓	
F8	Consideration of health predispositions	24% *	I	27% *	A		✓	✓		✓
F9	Health check/health diary	22% *	I	47% *	A		✓	✓	✓	✓
F10	Sharing data with doctors	8% *	A	2% ^2^	Mixed (A, O)					
F11	Sharing data with peers	3% ^2^	Mixed (I, R)	17% *	I			✓		
F12	Sharing data with organizations	4% ^2^	Mixed (R, I)	36% *	A					
F13	Communication with caregivers	8% *	I	58% *	A		✓		✓	✓
F14	Community forum	15% *	I	36% *	A					
F15	Social media	56% *	R	2% ^2^	Mixed (R, I)		✓			
F22	Virtual assistant	14% *	I	47% *	A			✓	✓	✓
F23	Health rewards	14% *	I	24% *	A					
						∑	4	4	6	5

Legend: * = Categorization according to Fong’sapproachLegend: ^1^ = (O + A + M) < > (I + R + Q) rule applicableLegend: ^2^ = (O + A + M) < > (I + R + Q) rule not applicableLegend: A = Attractive quality (delighter)Legend: O = One-dimensional quality (performance need)M = Must-be quality (basic need)I = Indifferent qualityR = Reverse quality

The comparison for all features and subsamples demonstrates the explanatory power of all the user characteristics for 9 of the 14 differently evaluated features (F2, F5, F7, F8, F9, F11, F13, F15, and F22). Therefore, the results support the hypotheses regarding the influences of privacy concerns (H2b), mHealth literacy (H3b), mHealth self-efficacy (H4b), and adult playfulness (H5b) on the evaluation of *some* of the PHR features. For five of the features, explanations via at least two user characteristics (F2, F8, F9, F13, and F22) indicate that the influences are not mutually exclusive. However, the comparison does not yield explanatory results for all features, implying that further explanatory factors may influence different evaluations of PHR features in the two investigated countries.

## Discussion

This study was motivated by two questions regarding *how* users across different countries evaluate specific features of mHealth apps and *whether* individual user characteristics can explain potential differences in evaluating these features. To answer the research questions and test the developed hypotheses, we conducted an online survey in Germany and Denmark and used PHRs as a prominent example of mHealth apps.

To answer the first research question, we composed a current and comprehensive list of 26 PHR features based on extant literature in the research stream of PHR functionalities and features. Further, we analyzed the evaluation of these features by potential German and Danish users. Using the Kano method, we empirically captured users’ perceptions of the PHR features as having an attractive, one-dimensional, must-be, indifferent, or reverse quality and found support for a multi-categorial structure of potential user satisfaction in both the German and Danish subsamples (H1). We found a nuanced situation where each of the different quality perceptions appears, and both cross-country similarities and differences exist.

To the best of our knowledge, our study is the first to include an evaluation of PHR features based on potential users’ perceptions; thus, we contribute to the overall understanding of PHR user satisfaction. For both countries, we demonstrated that certain PHR features are evaluated differently, indicating differences between Germans and Danes. Our study contributes to the extant cross-country research of categorization results based on the Kano method, which has repeatedly found differences of product features in the evaluation across different countries (e.g., Basfirinci & Mitra, 2015; Bennur & Jin, 2013; Hejaili et al., 2009). Further, we identified two especially interesting patterns, as they support Kano’s lifecycle theory (Kano, 2001). Because Denmark already launched PHRs in 2003, whereas Germany has not yet done so, one might expect that the Danish assessment is more mature than the German assessment. However, given the differences in user characteristics that extend beyond healthcare (e.g., privacy concerns), we do not assume that the evaluation of PHR features from a German user’s perspective would be identical to the current evaluation from a Danish user’s perspective.

Addressing the second research question, we collected data on four user characteristics: privacy concerns, mHealth literacy, mHealth self-efficacy, and adult playfulness. We found support for the hypotheses regarding significant cross-country differences. Compared to Danes, Germans tend to have higher privacy concerns (H2a), lower mHealth literacy (H3a), lower mHealth self-efficacy (H4a), and lower adult playfulness (H5a). While the results of the first three characteristics support the hypotheses, the significant difference regarding adult playfulness is revealing. It may be considered a complement to international adult playfulness and gamification research (Pang & Proyer, 2018).

Furthermore, we also present an approach to explain the differences in the feature evaluation with user characteristics. In this, we found support for the hypotheses concerning the explanatory power of user characteristics regarding feature evaluation, that is privacy concerns (H2b), mHealth literacy (H3b), mHealth self-efficacy (H4b), and adult playfulness (H5b) influence the evaluation of some PHR features. These cross-country differences in user characteristics may partly explain the cross-country differences in PHR feature evaluation for 9 out of 14 features with a cross-country difference. The extant literature applying the Kano method in health care (e.g., Materla et al., 2019) and other domains (e.g., Luor et al., 2015) focuses on the evaluation results without examining the underlying rationale behind the outcomes. Instead, this approach offers a new perspective of understanding differences in the evaluation and enriches the existing body of knowledge.

### Theoretical contributions

This work offers two key theoretical contributions, one for mHealth and one for Kano research. First, by applying the Kano method to evaluate PHR features, the results explain the relationship between certain PHR features and user satisfaction, building a bridge between more technical, feature-oriented mHealth research and more behavioral user acceptance and marketing-oriented mHealth research. Although other researchers have repeatedly demanded the application of the Kano model within the healthcare domain in general (Materla et al., 2019) and the evaluation of PHRs in particular (Baird et al., 2011), prior literature has lacked adequate examination of PHRs or other mHealth apps in connection with the satisfaction of potential users. Our work provides the first empirical arguments regarding which features can satisfy potential PHR users in the future. This can be a starting point for investigating other types of mHealth apps.

Second, using theoretical arguments and empirical evidence on the explanatory power of user characteristics regarding differences in the feature evaluation of Germans and Danes, we provide a methodological augmentation of the Kano method that can be applied to explain potential subgroup differences. The gathered knowledge associated with these differences can provide a starting point for further conceptual developments of the Kano method. Future studies applying the Kano method could collect data on other pertinent user characteristics that may influence the evaluation of product features. Our work is the first step toward understanding evaluation differences in the context of digitalized healthcare and, thus, may be used for the evaluation of other apps in health care and other domains.

### Managerial implications

Our work provides implications for mHealth app developers and policymakers. First, our work offers an up-to-date overview of potential PHR features that app developers can use as a starting point. Second, we learned that these features contribute differently to the satisfaction of potential users. App developers could use user perceptions to elaborate on where to invest resources in the future. Third, the results indicate the explanatory power of user characteristics regarding the evaluation of such features. Therefore, internationally operating app providers should be aware of country-specific differences and provide customizability regarding their respective solutions’ features.

Moreover, the results provide insight for policymakers. First, policymakers in Germany and Denmark could use user characteristics to educate their citizens or inform and consciously address potential users’ fears. Striving for user satisfaction could be the first step to increase the currently low adoption and retention rates of mHealth solutions significantly. Second, our study indicates major differences between the user characteristics in Germany and Denmark. Therefore, European policymakers in the healthcare domain could consider these differences in future European legislation, for example, by updating the existing EU legal framework applicable to lifestyle and wellbeing apps.

### Limitations

As in every research endeavor, our work has limitations. First, we focused solely on PHR as a major and potent yet single class of mHealth apps. Second, the literature review led to a comprehensive but not necessarily exhaustive set of PHR features. Other reviews and approaches might yield different features. Third, the set of PHR features was evaluated solely from a user’s point of view. Unlike other researchers who chose a clinical point of view within their studies (Hankins et al., 2020; Jongerius et al., 2019), we did not examine the importance of single PHR features from a clinical or organizational perspective within our study. Furthermore, our user-centric study contributes only indirectly to the important field of mHealth app regulation that is discussed by several other authors due to to the plethora of available mHealth apps (Larson, 2018; Rojas Mezarina et al., 2020). Fourth, we identified potential explanations for several differences in the feature evaluation based on user characteristics. However, some evaluation differences cannot be explained by the user characteristics covered in this study. There are likely other characteristics that we did not measure. For instance, users’ general experience of mHealth apps usage as well as other aspects such as time and support might be different in Germany and Denmark and could explain existing evaluation differences. Last, the empirical results’ generalizability is limited, and the results should only be interpreted in a country- and user-specific manner. Although we cover a broad range of sociodemographic characteristics, including different ages, educational backgrounds, and employment states, the sample is not representative of Germany or Denmark. Although our chosen methodological approach provides the highest possible degree of validity and reliability, the risk of bias cannot be completely excluded, due to the comparatively unbalanced sample and the overall small sample size. Furthermore, because most participants were not experienced using mHealth apps, the results only account for user evaluations in the preadoption stage. Future surveys and analyses must be conducted o verify the validity of the conclusions for other countries and user groups.

### Future research

Three promising directions for future research emerged from this work. First, due to the high speed of technological developments, future research could include new trends (e.g., augmentation or robotics in healthcare) and resulting features to have them evaluated in due course. An investigation of additional features could enrich the understanding of satisfaction drivers regarding PHRs. Second, we suggest expanding the scope of other potential explanatory user characteristics to increase future analyses’ power. We covered four pertinent user characteristics, although more research is still to be done. One promising direction for further user characteristics might be users’ general experience or exposure of mHealth apps usage or other influencing factors such as time and support. Additionally, this may also apply to non-covered user segments, as the sample data is not representative for Germany or Denmark. Finally, future research could focus on evaluating the general validity of our research in other countries, with other user groups, and other mHealth apps. More empirical research would help refine the identified influences of user characteristics and provide a better overall understanding of the relationships between user characteristics and the evaluation of PHR features. A first promising approach would be to focus on users that continually use PHRs or other mHealth apps.

## Conclusion

This study contributes to mHealth research by providing two novel results. First, using PHRs as an example, the application of the Kano method implies that app features contribute differently to the satisfaction of potential mHealth app users. We determine different influences on potential users’ satsifaction across a comprehensive list of 26 features and differences in the general perception in two countries. Second, our empirical study demonstrates significant differences between Germany and Denmark for all four user characteristics tested within our research. We found that Germans tend to have higher privacy concerns, lower mHealth literacy, lower mHealth self-efficacy, and lower adult playfulness than Danes. Moreover, we found that these differences in user characteristics explain some of the differences in evaluating distinct features. Thus, this paper contributes to a better understanding of what constitutes and influences user satisfaction concerning potential mHealth app features. We hope our findings regarding feature evaluation and user characteristics’ explanatory power stimulate further empirical studies on PHRs and other mHealth apps. Because this model implies application in two countries, it could be applied by global app providers in other countries to understand user needs better. Moreover, healthcare providers could apply the model when introducing or changing existing technical mHealth app solutions. Thus, our work may increase the adoption rates of existing and other promising mHealth solutions in the future.

## Appendix 1 Measures

All multi-item scales used 7-point measures. The scale anchors were 1: Strongly disagree – 7: Strongly agree.

*Privacy concerns* were calculated by averaging the four first-order construct scores of *collection*, *errors*, *unauthorized access* and *secondary use,* which were measured by adapting Angst and Agarwal’s (2009) fifteen-item scale*: Collection*: (1) It usually bothers me when healthcare entities ask me for personal information, (2) When healthcare entities ask me for personal information, I sometimes think twice before providing it, (3) It bothers me to give personal information to so many healthcare entities, (4) I am concerned that healthcare entities are collecting too much personal information about me. *Errors*: (1) All the personal information in computer databases should be double-checked for accuracy – no matter how much this costs, (2) Healthcare entities should take more steps to make sure that the personal information in their files is accurate, (3) Healthcare entities should have better procedures to correct errors in personal information, (4) Healthcare entities should devote more time and effort to verifying the accuracy of the personal information in their databases. *Unauthorized Access*: (1) Healthcare entities should devote more time and effort to preventing unauthorized access to personal information, (2) Computer databases that contain personal information should be protected from unauthorized access no matter how much it costs, (3) Healthcare entities should take more steps to make sure that unauthorized people cannot access personal information in their computers. *Secondary Use*: (1) Healthcare entities should not use personal information for any purpose unless it has been authorized by the individuals who provided the information, (2) When people give personal information to a company for some reason, the company should never use the information for any other reason, (3) Healthcare entities should never sell the personal information in their computer databases to other healthcare entities, (4) Healthcare entities should never share personal information with other healthcare entities unless it has been authorized by the patient who provided the information.

*mHealth literacy* was calculated by averaging the two first-order construct scores of *mHealth information searching* and *mHealth information appraisal*, which were measured by adapting Lin and Bautista’s (2017) eight-item scale: *mHealth information searching*: (1) I know how to find helpful health resources on the mobile phone, (2) I know how to use the mobile phone to answer my health questions, (3) I know what health resources are available on the mobile phone, (4) I know where to find helpful health resources on the mobile phone. *mHealth information appraisal*: (1) I know how to use the health information I find on the mobile phone to help me (dropped), (2) I have the skills I need to evaluate the health resources I find on the mobile phone, (3) I can tell high quality from low-quality health resources on the mobile phone, (4) I feel confident in using information from the mobile phone to make health decisions.

*mHealth self-efficacy* was measured using three items developed by Fox and Connolly (2018): (1) I could use health technologies to manage my health, if I had used a similar technology before, (2) I could use health technologies to manage my health, if someone showed me how to, (3) I could use health technologies to manage my health, if I had time to try them out.

*Adult playfulness* was measured using Proyer’s (2012) five-item scale: (1) I am a playful person, (2) Good friends would describe me as a playful person, (3) I frequently do playful things in my daily life, (4) It does not take much for me to change from a serious to a playful frame of mind, (5) Sometimes, I completely forget about the time and am absorbed in a playful activity.

**Table 9:** Factor Loadings from Exploratory Factor Analysis (main loading bold font)

	Factor							
Item	1	2	3	4	5	6	7	8
AdultPlayfulness1.	**0.95**	0.01	−0.06	−0.04	0.03	−0.04	0.07	0.02
AdultPlayfulness2.	**0.88**	−0.06	0.00	−0.08	−0.03	−0.02	0.05	0.02
AdultPlayfulness3.	**0.81**	−0.11	0.06	0.04	−0.05	−0.02	−0.04	0.03
AdultPlayfulness5.	**0.62**	0.09	0.09	−0.05	0.02	0.10	−0.04	−0.09
AdultPlayfulness4.	**0.60**	0.04	−0.10	0.07	0.03	−0.03	−0.03	0.02
mHealthLiteracy.InformationSearching4.	−0.03	**1.07**	−0.01	0.02	0.05	0.01	−0.10	−0.17
mHealthLiteracy.InformationSearching1.	−0.01	**0.76**	−0.01	−0.02	0.03	0.00	0.03	0.10
mHealthLiteracy.InformationSearching2.	0.05	**0.75**	−0.07	0.00	−0.07	−0.01	0.17	0.05
mHealthLiteracy.InformationSearching3.	−0.04	**0.74**	0.08	0.00	−0.03	−0.01	−0.08	0.04
PrivacyConcerns.Collection1.	−0.02	0.02	**0.87**	−0.07	−0.04	−0.06	0.02	0.04
PrivacyConcerns.Collection2.	−0.05	0.02	**0.82**	0.07	0.01	0.01	−0.10	0.00
PrivacyConcerns.Collection3.	0.00	0.01	**0.82**	−0.10	−0.01	0.03	0.14	−0.02
PrivacyConcerns.Collection4.	0.04	−0.05	**0.79**	0.07	0.09	−0.02	−0.02	0.00
PrivacyConcerns.Errors4.	−0.03	0.02	−0.02	**0.92**	0.00	−0.02	−0.06	−0.03
PrivacyConcerns.Errors2.	0.03	0.02	−0.05	**0.89**	−0.03	−0.04	0.02	0.02
PrivacyConcerns.Errors3.	−0.06	0.00	−0.11	**0.83**	0.07	0.00	0.00	0.04
PrivacyConcerns.Errors1.	0.03	−0.04	0.22	**0.58**	−0.09	0.08	0.09	−0.07
PrivacyConcerns.SecondaryUse1.	0.02	0.05	0.09	0.03	**0.78**	0.07	−0.12	−0.02
PrivacyConcerns.SecondaryUse4.	0.02	−0.03	0.09	0.04	**0.75**	−0.07	−0.07	0.07
PrivacyConcerns.SecondaryUse3.	−0.05	0.02	−0.07	−0.06	**0.65**	0.01	0.19	−0.02
PrivacyConcerns.SecondaryUse2.	0.01	−0.06	−0.07	−0.01	**0.64**	0.00	0.10	−0.05
mHealthSelf-Efficacy2.	−0.04	−0.05	0.02	0.02	−0.02	**0.92**	−0.03	−0.04
mHealthSelf-Efficacy3.	−0.04	−0.06	−0.03	−0.05	0.04	**0.85**	0.06	0.10
mHealthSelf-Efficacy1.	0.08	0.10	−0.03	0.02	−0.01	**0.72**	−0.02	0.01
PrivacyConcerns.UnauthorizedAccess3.	−0.05	0.04	0.02	0.03	0.07	0.04	**0.86**	−0.02
PrivacyConcerns.UnauthorizedAccess2.	0.01	−0.01	0.02	−0.02	0.04	−0.03	**0.79**	−0.02
PrivacyConcerns.UnauthorizedAccess1.	0.04	−0.01	0.03	0.23	0.07	0.01	**0.60**	0.03
mHealthLiteracy.InformationAppraisal3.	−0.04	0.06	0.03	−0.06	−0.05	0.01	0.06	**0.85**
mHealthLiteracy.InformationAppraisal4.	−0.01	0.00	−0.02	0.00	0.02	0.10	−0.10	**0.74**
mHealthLiteracy.InformationAppraisal2.	0.07	0.20	0.05	0.07	0.02	−0.07	0.02	**0.69**
Eigenvalue	**3.09**	**3.00**	**2.86**	**2.84**	**2.15**	**2.14**	**2.06**	**1.95**
Percentage of variance explained (%)	**10.30**	**10.01**	**9.54**	**9.45**	**7.18**	**7.14**	**6.86**	**6.49**

**Table 10:** Confirmatory factor analysis fit measures

Fit measure	Value	Level of acceptance	Reference
	662.156/377.000 = 1.76	< 3	Wheaton et al. (1977)
CFI	0.942	> 0.9	Bentler (1990)
TLI	0.934	> 0.9	Tucker and Lewis (1973)
AGFI	0.826	> 0.8	Jöreskog and Sörbom (1986)
RMSEA	0.053	< 0.6	Steiger (1980)

## Appendix 3 Sample characteristics

**Table 11:** Sample characteristics

Variable	Germany (n = 215)		Denmark (n = 59)		Entire sample (n = 274)	
	absolute	relative	absolute	relative	absolute	relative
Gender						
female	100	47%	32	54%	132	48%
male	115	53%	27	46%	142	52%
Age						
18–25	97	45%	19	32%	116	42%
26–35	95	44%	21	36%	116	42%
36–45	11	5%	12	20%	23	8%
46–73	12	6%	7	12%	19	7%
Employment status						
Student	115	53%	24	41%	139	51%
Employed	93	43%	33	56%	126	46%
Self-employed	2	1%	1	2%	3	1%
Unemployed	1	0%	1	2%	2	1%
Retired	4	2%	0	0%	4	1%
Educational background						
Less than a high school diploma	3	1%	0	0%	3	1%
High school degree or equivalent	34	16%	8	14%	42	15%
Bachelor’s degree or equivalent	82	38%	33	56%	115	42%
Master’s degree or equivalent	83	39%	13	22%	96	35%
Doctoral degree or equivalent	13	6%	5	8%	18	7%
Usage of healthcare-related apps						
Never	103	48%	21	36%	124	45%
Less than once a month	47	22%	26	44%	73	27%
More than once a month	24	11%	10	17%	34	12%
Once a week	16	7%	0	0%	16	6%
More than once a week	6	3%	1	2%	7	3%
Daily	19	9%	1	2%	20	7%

To compare the cultural values of Germans and Danes, we used the Values Survey Module questions (Hofstede et al., 2013) covering the six Hofstede dimensions (Hofstede et al., 2010). For calculating the constant, we chose Denmark as reference and see that the differences between our samples are qualitatively the same as the differences between the countries’ cultures in the data of Hofstede Insights (2020).

**Table 12:** Cultural dimensions of participants in Germany and Denmark

	Country	Power Distance	Individualism	Masculinity	Uncertainty Avoidance	Long Term Orientation	Indulgence
Scores according to Hofstede	Denmark	18	74	16	23	35	70
	Germany	35	67	66	65	83	40
Difference according to Hofstede (Germany minus Denmark)		+17	−10	+50	+42	+38	−30
Scores in our survey	Denmark	18	74	16	23	35	70
	Germany	40	66	60	50	63	43
Difference in our data (Germany minus Denmark)		+22	−8	+44	+27	+28	−27

## Appendix 4 Sample split

**Table 13:** Empirical results of the PHR feature evaluation based on the sample split

	#	1st quartile (“low”)								2nd and 3rd quartile (“middle”)								4th quartile (“high”)							
		A	O	M	I	R	Q	Category strength	Category	A	O	M	I	R	Q	Category strength	Category	A	O	M	I	R	Q	Category strength	Category
Privacy Concerns	F2	45%	21%	10%	15%	7%	1%	24% *	A	30%	29%	15%	23%	4%	0%	1% ^2^	Mixed (A, O, I)	25%	15%	12%	33%	15%	0%	7% ^1^	A
	F4	31%	31%	10%	25%	3%	0%	0% ^2^	Mixed (A, O)	16%	30%	19%	30%	4%	1%	0% ^1^	O	19%	22%	15%	30%	13%	0%	7% ^1^	O
	F5	39%	24%	11%	21%	4%	0%	15% *	A	38%	23%	6%	26%	7%	0%	11% *	A	22%	22%	4%	33%	18%	0%	10% ^1^	Mixed (A, O, I)
	F7	41%	21%	7%	31%	0%	0%	10% ^1^	A	35%	29%	10%	26%	1%	0%	6% ^2^	Mixed (A, O, I)	36%	24%	12%	19%	9%	0%	12% ^2^	Mixed (A, O, R, M, I)
	F8	46%	4%	3%	38%	8%	0%	8% ^1^	A	31%	7%	1%	49%	10%	1%	18% *	I	22%	1%	6%	49%	21%	0%	27% *	R
	F9	41%	7%	6%	38%	8%	0%	3% ^1^	A	33%	14%	0%	43%	10%	0%	10% ^1^	I	28%	4%	1%	40%	25%	0%	12% ^1^	R
	F10	41%	27%	13%	18%	1%	0%	14% *	A	32%	25%	18%	21%	5%	0%	7% ^2^	Mixed (A, O, I)	27%	22%	16%	16%	18%	0%	4% ^2^	Mixed (R, I)
	F11	17%	3%	3%	54%	24%	0%	30% *	I	11%	1%	1%	51%	35%	1%	15% *	I	10%	1%	0%	34%	54%	0%	19% *	R
	F12	28%	6%	0%	39%	27%	0%	11% ^1^	I	22%	3%	2%	37%	35%	1%	1% ^2^	Mixed (I, R)	9%	1%	1%	28%	60%	0%	31% *	Mixed (I, R, A)
	F13	56%	10%	3%	28%	3%	0%	28% *	A	41%	10%	2%	43%	4%	0%	2% ^1^	A	36%	12%	1%	37%	13%	0%	1% ^1^	A
	F14	35%	6%	0%	39%	20%	0%	4% ^1^	I	26%	6%	1%	44%	22%	1%	18% *	I	22%	0%	0%	37%	40%	0%	3% ^2^	O
	F15	3%	0%	0%	42%	55%	0%	13% ^2^	Mixed (R, I)	1%	1%	0%	22%	76%	1%	54% *	R	0%	0%	0%	21%	79%	0%	58% *	Mixed (A, O, I)
	F22	45%	4%	0%	42%	8%	0%	3% ^1^	I	38%	8%	1%	43%	10%	0%	5% ^1^	I	42%	1%	0%	39%	18%	0%	3% ^1^	Mixed (A, O, R, M, I)
	F23	35%	6%	1%	35%	23%	0%	0% ^1^	A	29%	1%	0%	43%	26%	0%	13% *	I	25%	1%	0%	42%	31%	0%	10% ^2^	R
mHealth Literacy	F2	26%	27%	11%	27%	9%	0%	0% ^1^	O	39%	18%	14%	23%	7%	0%	16% *	A	28%	30%	12%	21%	7%	1%	1% ^2^	Mixed (O, A, I)
	F4	16%	31%	11%	34%	7%	0%	3% ^1^	O	23%	26%	15%	29%	6%	1%	3% ^1^	O	21%	30%	22%	22%	4%	0%	7% ^2^	Mixed (O, M, I, A)
	F5	31%	23%	7%	30%	9%	0%	1% ^1^	A	36%	20%	4%	30%	9%	0%	7% ^1^	A	33%	30%	12%	16%	9%	0%	3% ^2^	Mixed (A, O)
	F7	39%	30%	7%	24%	0%	0%	9% ^2^	Mixed (A, O, I)	39%	20%	10%	27%	3%	0%	12% *	A	28%	31%	10%	25%	4%	0%	3% ^2^	Mixed (O, A, I)
	F8	27%	7%	3%	44%	17%	1%	17% *	I	31%	2%	3%	50%	12%	1%	19% *	I	42%	9%	3%	39%	7%	0%	3% ^1^	A
	F9	26%	11%	1%	44%	17%	0%	19% *	I	32%	9%	3%	42%	14%	0%	9% ^1^	I	46%	9%	0%	36%	9%	0%	10% ^1^	A
	F10	30%	29%	11%	21%	9%	0%	1% ^2^	Mixed (A, O, I)	36%	20%	18%	20%	6%	0%	16% *	A	28%	30%	18%	15%	9%	0%	1% ^2^	Mixed (O, A, M, I)
	F11	7%	3%	3%	50%	37%	0%	13% ^2^	Mixed (I, R)	12%	1%	0%	47%	39%	1%	7% ^2^	Mixed (I, R)	19%	1%	1%	46%	31%	0%	15% *	I
	F12	19%	1%	0%	40%	40%	0%	0% ^2^	Mixed (I, R)	19%	3%	1%	34%	42%	1%	7% ^2^	Mixed (R, I)	25%	6%	3%	33%	33%	0%	0% ^2^	Mixed (I, R, A)
	F13	33%	11%	6%	41%	9%	0%	9% ^1^	A	48%	9%	1%	37%	4%	0%	11% *	A	46%	12%	0%	36%	6%	0%	10% ^1^	A
	F14	30%	6%	0%	37%	26%	1%	7% ^1^	I	23%	4%	0%	47%	26%	0%	20% *	I	36%	3%	1%	34%	25%	0%	1% ^1^	I
	F15	3%	0%	0%	27%	70%	0%	43% *	R	1%	0%	0%	27%	72%	1%	45% *	R	0%	1%	0%	27%	72%	0%	45% *	R
	F22	37%	6%	1%	43%	13%	0%	6% ^1^	I	39%	6%	0%	45%	11%	0%	6% ^1^	I	49%	4%	0%	36%	10%	0%	13% ^1^	A
	F23	26%	4%	0%	39%	31%	0%	7% ^2^	Mixed (I, R, A)	28%	2%	1%	45%	24%	0%	16% *	I	37%	1%	0%	34%	27%	0%	3% ^1^	I
mHealth Self-Efficacy	F2	24%	15%	17%	30%	14%	0%	6% ^1^	A	39%	25%	9%	23%	4%	0%	13% *	A	33%	30%	14%	16%	5%	2%	3% ^2^	Mixed (A, O)
	F4	17%	19%	14%	39%	11%	0%	20% *	I	25%	31%	10%	30%	3%	1%	1% ^1^	O	17%	37%	29%	13%	5%	0%	8% ^2^	Mixed (O, M)
	F5	27%	17%	7%	33%	15%	0%	6% ^1^	A	42%	20%	7%	25%	6%	0%	17% *	A	29%	37%	6%	21%	8%	0%	8% ^2^	Mixed (O, A, I)
	F7	31%	24%	11%	31%	4%	0%	0% ^1^	A	39%	24%	8%	27%	2%	0%	13% *	A	38%	30%	11%	17%	3%	0%	8% ^2^	Mixed (A, O)
	F8	21%	6%	2%	49%	19%	2%	27% *	I	35%	5%	5%	47%	9%	0%	13% *	I	44%	5%	0%	40%	11%	0%	5% ^1^	I
	F9	20%	7%	2%	49%	21%	0%	27% *	I	35%	13%	1%	39%	12%	0%	4% ^1^	I	49%	8%	3%	33%	6%	0%	16% *	A
	F10	29%	21%	15%	21%	13%	0%	7% ^2^	Mixed (A, O, I, M)	33%	23%	16%	23%	6%	0%	10% *	A	38%	33%	17%	8%	3%	0%	5% ^2^	Mixed (A, O)
	F11	6%	2%	2%	38%	51%	0%	13% *	R	17%	2%	1%	50%	30%	1%	20% *	I	13%	2%	0%	54%	32%	0%	22% *	I
	F12	11%	1%	1%	31%	56%	0%	25% *	R	21%	5%	2%	40%	31%	1%	9% ^2^	Mixed (I, R)	32%	3%	2%	32%	32%	0%	0% ^1^	I
	F13	33%	10%	2%	44%	11%	0%	11% ^1^	I	46%	9%	2%	38%	6%	0%	9% ^1^	A	52%	14%	3%	30%	0%	0%	22% *	A
	F14	18%	6%	0%	44%	32%	0%	12% ^2^	Mixed (I, R)	33%	3%	1%	39%	24%	1%	6% ^1^	I	30%	5%	0%	43%	22%	0%	13% ^1^	I
	F15	1%	0%	0%	19%	80%	0%	61% *	R	2%	1%	0%	33%	64%	1%	31% *	R	0%	0%	0%	25%	75%	0%	49% *	R
	F22	31%	5%	1%	48%	15%	0%	17% *	I	40%	8%	0%	43%	9%	0%	3% ^1^	I	56%	2%	0%	32%	11%	0%	24% *	A
	F23	26%	2%	0%	44%	27%	0%	17% *	I	33%	2%	1%	39%	26%	0%	6% ^1^	I	29%	5%	0%	40%	27%	0%	11% ^1^	I
Playfulness	F2	42%	26%	8%	18%	6%	0%	17% *	A	29%	20%	17%	27%	6%	0%	2% ^1^	A	30%	27%	9%	22%	11%	2%	3% ^2^	Mixed (A, O, I)
	F4	17%	33%	17%	27%	5%	1%	6% ^1^	O	28%	20%	14%	33%	5%	0%	5% ^1^	A	11%	41%	17%	23%	8%	0%	17% *	O
	F5	36%	22%	5%	28%	9%	0%	8% ^1^	A	38%	18%	8%	26%	11%	0%	12% *	A	25%	34%	8%	27%	6%	0%	8% ^1^	O
	F7	40%	28%	10%	21%	1%	0%	12% ^2^	Mixed (A, O)	39%	17%	8%	35%	2%	0%	4% ^1^	A	28%	41%	13%	14%	5%	0%	13% ^2^	Mixed (O, A)
	F8	36%	8%	3%	41%	13%	0%	5% ^1^	I	28%	2%	3%	53%	12%	2%	25% *	I	39%	8%	3%	38%	13%	0%	2% ^1^	A
	F9	26%	9%	3%	50%	13%	0%	24% *	I	35%	8%	2%	41%	14%	0%	6% ^1^	I	42%	14%	0%	30%	14%	0%	13% ^1^	A
	F10	33%	22%	18%	21%	6%	0%	12% ^2^	Mixed (A, O, I)	37%	23%	11%	23%	6%	0%	14% *	A	23%	31%	25%	9%	11%	0%	6% ^2^	Mixed (O, M, A)
	F11	13%	4%	3%	46%	35%	0%	12% ^2^	Mixed (I, R)	9%	1%	0%	50%	39%	1%	11% *	I	19%	2%	2%	44%	34%	0%	9% ^2^	Mixed (I, R)
	F12	23%	4%	3%	38%	32%	0%	6% ^2^	Mixed (I, R, A)	16%	2%	1%	37%	44%	1%	7% ^2^	Mixed (R, I)	27%	6%	2%	28%	38%	0%	9% ^2^	Mixed (R, I, A)
	F13	42%	4%	1%	45%	8%	0%	3% ^1^	I	39%	13%	2%	41%	6%	0%	2% ^1^	A	56%	13%	5%	23%	3%	0%	33% *	A
	F14	31%	3%	0%	38%	28%	0%	8% ^1^	I	24%	5%	0%	45%	25%	1%	20% *	I	31%	6%	2%	36%	25%	0%	5% ^1^	R
	F15	3%	0%	0%	23%	74%	0%	51% *	R	0%	0%	0%	31%	68%	1%	37% *	R	2%	2%	0%	23%	73%	0%	50% *	A
	F22	35%	6%	1%	46%	12%	0%	12% ^1^	I	38%	5%	0%	45%	11%	0%	8% ^1^	I	55%	5%	0%	30%	11%	0%	25% *	R
	F23	22%	1%	0%	49%	28%	0%	21% *	I	30%	2%	1%	42%	25%	0%	13% *	I	41%	5%	0%	27%	28%	0%	13% ^1^	Mixed (A, O, I)

Legend: * = Categorization significant at a ten-percent level according to Fong test

Legend: ^1^ = (O + A + M) < > (I + R + Q) rule applicable

Legend: ^2^ = (O + A + M) < > (I + R + Q) rule not applicable

A = Attractive quality (delighter)

O = One-dimensional quality (performance need)

M = Must-be quality (basic need)

I = Indifferent quality

R = Reverse quality

Q = Questionable result

## Footnotes

1 We define country affiliation by the country in which the study participants have spent most of their life.

2 Market research usually refers to *customer satisfaction*. Because this work examines an mHealth app, we use the term *user satisfaction*.

3 The term *Kano method* refers to the procedure (i.e., the questioning technique) for categorizing features and for different evaluation rules. The term *Kano model* refers to the concept of customer satisfaction as presented in the previous chapter.
